# Anxiety-depressive disorders in patients with dysarthria against the background of organic brain damage

**DOI:** 10.1192/j.eurpsy.2021.1829

**Published:** 2021-08-13

**Authors:** I. Mudrenko, O. Kolenko

**Affiliations:** Department Of Neurosurgery And Neurology, Sumy State University, Sumy, Ukraine

**Keywords:** dysarthria, anxiety, depression, cerebrovascular accident

## Abstract

**Introduction:**

The presence of acquired speech disorders of varying evidence can cause maladjustment and job loss. Often there is no adequate psychological and psychotherapeutic assistance for these patients, which hinders the process of recovery and reintegration into the social environment.

**Objectives:**

To study the level of anxiety and depression in patients with dysarthria who have undergone various types of cerebrovascular accidents. To give practical recommendations regarding the correction of these conditions.

**Methods:**

To assess the level of anxiety and depression, the Hospital Anxiety and Depression Scale (HADS) was used as the most convenient for application in clinical practice.

**Results:**

The study involved 42 people in the age group 45-60 years old with the consequences of cerebrovascular accident in the form of various types of dysarthria and without severe movement disorders. All participants had a university degree and a confirmed stroke of anamnesis. According to the data obtained, 45% of patients had symptoms of depression, 52% – anxiety. It should be clarified that specific weight of men with manifestations of depression and anxiety was higher (65% and 56%, respectively). The beginning of active antidepressant therapy in a hospital setting showed a positive subjective effect from such influences – in 38% of patients.

**Conclusions:**

The use of modern methods for assessing the level of anxiety and depression in patients with speech disorders should become an obligatory stage of diagnostic measures. Psychological assistance and pharmacological correction not only helps patients to adapt to new social conditions, but also promotes prevention the progression of depressive manifestations.

**Disclosure:**

No significant relationships.

